# The Wages of Original Antigenic Sin

**DOI:** 10.3201/eid1606.100453

**Published:** 2010-06

**Authors:** David M. Morens, Donald S. Burke, Scott B. Halstead

**Affiliations:** National Institutes of Health, Bethesda, Maryland, USA, and Associate Editor, Emerging Infectious Diseases (D.M. Morens); University of Pittsburgh Graduate School of Public Health, Pittsburgh, Pennsylvania, USA, and former Editorial Board member, Emerging Infectious Diseases (D.S. Burke); Uniformed Services University of the Health Sciences, Bethesda, and Editorial Board, Emerging Infectious Diseases (S.B. Halstead)

**Keywords:** Influenza, antigenicity, H1N1, immunity, viruses, vaccine, pandemic, original antigenic sin, commentary

“The deliberate sin of the first man is the cause of original sin”

—[Saint] Augustine of Hippo, Algerian Christian theologian (354 ad–430 ad), De nuptiis et concupiscientia [On Marriage and Concupiscence], II, xxvi, 43

What epidemiologist Thomas Francis, Jr. (1900–1969) was thinking when pondering certain inexplicable serologic data from a 1946 influenza vaccine trial may never be known. Whether in religious reverence for the beauty of science or impish delight fueled by the martini breaks of which he was so fond, Francis coined the term “original antigenic sin” to describe a curious new immunologic phenomenon. Elsewhere in this issue, Adalja and Henderson propose that original antigenic sin has altered the population age–specific incidence of infection and disease caused by influenza A pandemic (H1N1) 2009 virus and that public health responses must account for the disruption (*1*). What is original antigenic sin, what is its immunologic basis, and into what sort of trouble is it getting us?

Discovery of influenza viruses in the early 1930s ignited a search to understand the epidemiology of pandemic/endemic influenza. Serologic data showed that decendents of the 1918 pandemic influenza virus were still circulating and were changing antigenically (we would now say drifting and undergoing intrasubtypic reassortment); that contemporary human and swine viruses were closely related; and that over a lifetime of repeated exposures, different human birth cohorts were acquiring fundamentally different influenza infection experiences. The surprise appearance in 1946 of a new and antigenically different influenza A virus (designated influenza A′ and recently shown to be a subtype H1N1 intrasubtypic reassortant) provided Francis a unique opportunity. College students participating in a 1946 trial of the old 1946 virus vaccine were infected in March 1947 with the new A′ virus. Surprisingly, these students developed low serologic titers to the new infecting virus and higher seroconverting titers to old viruses with which they previously had been infected. Moreover, recent recipients of the old virus vaccine had the highest seroconverting titers of all to the old—but not to the new—virus (*2**,**3*).

Absorption studies, in which various viruses were used to selectively remove serum antibodies, suggested that repeat exposures to dominant antigens of first-infecting viruses, when seen later as lesser or secondary antigens on subsequently infecting viruses, somehow reinforced antibody responses to the original strains at the apparent expense of responses to newer strains (*4*). Francis announced “the doctrine of original antigenic sin” (*5**,**6*): “[t]he antibody-forming mechanisms appear to be oriented by the initial infections of childhood so that exposures later in life to antigenically related strains result in a progressive reinforcement of the primary antibody” (*3*). Later studies by many investigators showed original antigenic sin to be a general phenomenon associated with numerous related/sequentially infecting virus strains that contain multiple external epitopes of varying cross-specificity (i.e., ability to elicit cross-reactive antibody), including antigenically drifting viruses such as influenza A, and the more stable flaviviruses, which circulate concurrently as multiple distinct viruses, virus serotypes, and virus strains (*7**,**8*).

Original antigenic sin seems to be most pronounced when sequential viruses are of intermediate antigenic relatedness; when they are antigenically complex; and when sequential exposure intervals are long, consistent with ongoing selection and expansion of lymphocyte clones that have increasing antibody avidity at key cross-reactive epitopes (*7**–**10*) and possibly with epitope competition between naïve and antigen-specific B cells (*8*). A phenomenon analogous to original antigenic sin also has been described with cytotoxic T lymphocytes (*11*). Although conclusive evidence in humans is lacking, original antigenic sin recently has come under scrutiny as a possible cause of viral immune escape, enhanced disease severity, decreased efficacy of influenza vaccines (*8,12–14*), and increased incidence of influenza in 2009 after vaccination with a related virus in 2008–2009 (*15*). On a positive note, original antigenic sin has also been linked to vaccine-induction of heterosubtypic neutralizing antibodies (*16*).

Adalja and Henderson note that the apparently lower incidence and severity of disease in older persons during the 2009–10 influenza pandemic probably reflects immunity to previously circulating influenza (H1N1) subtypes (*1*). Reichert et al. also attribute this age structure to original antigenic sin but emphasize the importance of exposures to the changing hemagglutinin glycosylation patterns of earlier influenza (H1N1) subtypes (e.g., those circulating before and after 1948) on a background of relatively conserved T-cell epitopes (*14*). However, the possibility that the age structure of pandemic (H1N1) 2009 infection is due simply to single or repeated exposures to different or differentially exposed hemagglutinin epitopes has not been ruled out. Useful information bearing on these questions might be gained by comparing antibody levels, antibody reactivities, and the original antigenic sin phenomenon in serum samples from the various age cohorts that had early exposures to markedly different (or to no) influenza (H1N1) serotypes, e.g., persons born before 1918; during 1918–1927, 1928–1946, 1947–1956, and 1957–1976; and after 1976. Of related interest are the 2009 influenza experiences of the ≈25.6 million persons living in America vaccinated with the 1976 Hsw1N1 vaccine (*17*), including 2.5 million born during 1957–1975, when influenza (H1N1) viruses did not circulate

The current pandemic provides the challenge to public health responses that Adjala and Henderson describe, as well as an opportunity to extend the efforts of Francis to better understand the complicated epidemiology of influenza. Is original antigenic sin really a sin from which our immune systems need to be saved? Or is it an epidemiologic blessing in disguise? We have much more to learn. As St. Augustine wrote (Confessiones, 8, 7): “Lord make me chaste—but not yet.”
